# Exploring Associations and Mediating Factors between Multiple Trace Metals with Anemia in US Adults: Insight from NHANES 2017–2020

**DOI:** 10.3390/nu16193424

**Published:** 2024-10-09

**Authors:** Lijie Xie, Xinchao Guan, Yixiang Zhou, Yujie He, Shilin Chen, Wanting Xiao, Jilong Yang, Jianyong Lu, Liecheng Hong, Qiansheng Hu, Qiong Wang, Chuanwen Li, Qing Wang

**Affiliations:** 1 School of Public Health, Sun Yat-sen University, Guangzhou 510080, China; 2 Public Health Service Center, Bao’an District, Shenzhen 518126, China

**Keywords:** anemia, trace metals, iron status, inflammation, NHANES

## Abstract

Background: Anemia significantly contributes to the global disease burden, with its incidence potentially influenced by the trace metal content within the body. Objective: This study aims to examine the associations between trace metals and anemia risk, with a particular focus on investigating the potential mediating roles of iron status and inflammation in these associations. Methods: Five trace metals (Ni, Co, Mn, Se, and Mo) were examined in 1274 US adults, utilizing data from the National Health and Nutrition Examination Survey (NHANES) 2017–2020. The individual and combined effects of these metals on anemia were assessed using logistic regression, quantile g-computation (QGC), and Bayesian kernel machine regression (BKMR). A sex-stratified analysis was conducted to discern any gender-specific susceptibilities. Additionally, mediation analysis was employed to explore the potential mediating roles of iron status and inflammation in the associations between these metals and anemia. Results: Increased risks of anemia were positively associated with Co and Ni levels but negatively correlated with Se and Mn levels (all with *p* < 0.05). The trace metal mixture was negatively associated with anemia, with the highest weights of Co and Se in different directions in both the QGC and BKMR models. In the sex-specific analysis, we observed less pronounced protective effects from trace metals in females. Moreover, the mediating proportion of the iron status and inflammation in these relationships ranged from 10.29% to 58.18%. Conclusion: Our findings suggest that the trace element mixture was associated with decreased anemia risk, among which Se was a protective factor while Co was a risk factor, and females were more susceptible. The effects of these trace metals on anemia may be mediated by the iron status and inflammation.

## 1. Introduction

Anemia is a significant global public health concern particularly affecting women and children, with its prevalence increasing among individuals over the age of 29 [1]. Anemia and its complications contribute to significant morbidity and mortality, further exacerbating the global burden of disease [2,3]. The etiology of anemia is multifactorial, with micronutrient deficiency, inherited hemoglobin disorders, as well as chronic and infectious diseases identified as the main risk factors [4,5]. Despite various interventions, such as dietary iron supplementation and public health initiatives, the prevalence of anemia remained alarmingly high in 2019, affecting approximately 1.8 billion people worldwide [6]. Therefore, it is imperative to identify novel risk factors for developing more effective anemia prevention strategies.

Emerging evidence suggests that imbalances in trace metals may also contribute to the increased prevalence of anemia. The health effects of these trace metals are complex and depend on maintaining optimal concentrations. Trace metals act as cofactors for numerous enzymes, playing crucial roles in antioxidant defense, metabolism, and immunological and neurological function [7,8]. However, excessive exposure to these metals—often caused by environmental pollution from sources such as industrial waste and mining activities [9,10]—can lead to adverse health effects. Additionally, insufficient dietary intake of these metals can contribute to or worsen health problems [7,11]. Emerging evidence has reported the associations between improper trace metal exposure and an increased risk of anemia [12,13,14]. Therefore, further investigation into the appropriate levels of these metals is necessary, particularly regarding the exposure–response relationships between trace metals and anemia. Research on this topic remains limited and presents inconsistent findings. A study on sub-Saharan immigrants indicated that the blood levels of trace metals, including Se, Mn, and Mo, were significantly lower in the anemic population, suggesting potential protective effects from these trace metals [15]. However, Qiao et al. found that several trace metals, including Mn, Se, and Co, were associated with increased risk of anemia, though no significance was found for Mo [13]. In contrast, Pan et al. reported a positive association between serum Se and hemoglobin levels, but a negative association with Mn [16]. Thus, further studies on the associations of trace metals with anemia are warranted.

Iron deficiency and inflammation are the predominant factors contributing to the development of anemia [17]. Iron deficiency occurs when there is insufficient dietary iron intake, malabsorption, or chronic blood loss, impairing the normal process of hemoglobin synthesis and ultimately leading to anemia [18]. Anemia of inflammation results from three major pathophysiological pathways: iron restriction, suppression of erythropoietic activity, and decreased erythrocyte survival [17]. Moreover, trace metals have been reported to be associated with both iron metabolism and inflammation. Gunshin et al. found that Mn, Ni, and Co can inhibit iron absorption by competitively binding to DMT1 [19]. Several epidemiological studies have demonstrated negative associations between these metals and the iron status [20,21,22,23]. Additionally, imbalances in trace metals can disrupt immune homeostasis and promote inflammatory responses [24,25]. Based on this evidence, we hypothesize that the iron status and inflammation may mediate the relationship between trace metals and the risk of anemia.

In conclusion, the current evidence on the association between trace metals and anemia is still limited and inconclusive, and the underlying mechanisms remain unclear. Therefore, we conducted this study to evaluate the association between trace metal exposure and the risk of anemia and to further assess the mediating role of the iron status and inflammation in these associations using data from the National Health and Nutrition Examination Survey (NHANES) 2017–2020.

## 2. Materials and Methods

### 2.1. Population

The NHANES is a series of nationally representative cross-sectional surveys conducted by the US Centers for Disease Control and Prevention (CDC). The survey protocol was approved by the Institutional Review Board of the National Center for Health Statistics. Data for this study were obtained from NHANES 2017–2020 (*n* = 15,560), which are publicly available on the CDC’s official website. In this survey cycle, 1793 participants had complete data on trace metals, hemoglobin, serum ferritin, albumin, and high-sensitivity C-reactive protein (hsCRP). We further excluded 519 individuals with missing covariates, including demographic information, body mass index (BMI), alcohol consumption, or measured blood pressure. A total of 1274 participants were included in the final analysis. The participant selection process is shown in Figure S1.

### 2.2. Assessment of Trace Metals and Mediators

Biological specimens, including blood, urine, and other samples, were collected at a mobile examination center and processed according to standardized protocols. The concentrations of the metals were measured using inductively coupled plasma dynamic reaction cell mass spectrometry (ICP-DRC-MS). A total of 7 metals in serum and 11 metals in urine were detected during the NHANES 2017–2020 cycle (Table S1). The reference values for these metals were obtained from the biomonitoring data reported by the US CDC and the previous literature [26,27,28]. Among these metals, six trace metals were identified: serum manganese (Mn), serum cobalt (Co), serum selenium (Se), serum chromium (Cr), urinary nickel (Ni), and urinary molybdenum (Mo). Since the other elements did not deviate from their reference values, we assumed they were not risk factors. Additionally, serum Cr was excluded to guarantee the reliability and stability of the results due to the low detection rate, and only Ni, Co, Mn, Se, and Mo were included in the final analyses for this study. For values below the limit of detection (LOD), the data were imputed as the LOD divided by the square root of 2. To account for dilution-dependent variation in urinary metals, the concentrations of Ni and Mo were adjusted based on the corresponding urinary creatinine levels.

Serum ferritin was measured using the Roche Cobas^®®^ e601(Roche Diagnostics, Indianapolis, IN, USA), employing a sandwich immunoassay with chemiluminescence detection. The serum albumin concentration was determined using the LX20 method, in which the dye bromocresol purple (BCP) selectively binds to albumin, causing a color change. High-sensitivity C-reactive protein (hsCRP) levels were determined using latex-enhanced nephelometry with particle-enhanced assays performed on a Behring Nephelometer for accurate measurement. Rigorous quality control procedures were implemented to ensure the accuracy and reliability of all measurements. Detailed laboratory procedures and quality control protocols are available on the NHANES website (https://www.cdc.gov/nchs/nhanes/index.htm, accessed on 15 May 2024).

### 2.3. Definition of Anemia

Anemia was defined based on the diagnostic guidelines proposed by the World Health Organization (WHO) [29]. Participants were classified as anemic if their hemoglobin (Hb) levels were below 110 g/L for pregnant women, 120 g/L for non-pregnant women, or 130 g/L for men.

### 2.4. Covariates

Covariates were selected based on the previous literature related to trace metal exposure or the risk of anemia [30,31]. These covariates included age, sex, race or ethnicity, body mass index (BMI, kg/m^2^), family poverty income ratio (PIR), physical activity, alcohol consumption, smoking status, diabetes mellitus, and hypertension. A PIR of less than 1 indicates that the family’s income is below the poverty level. Self-reported physical activity was categorized as “inactive” (<10 min per week) or “active” (≥10 min per week). Based on the self-reported drinking frequency, alcohol consumption was classified as “non-drinker”, “moderate drinker” (<1 time per week), or “heavy drinker” (≥1 time per week). According to the serum cotinine and self-reported smoking frequency [32], participants were classified as (1) “active smoker” if they had a serum cotinine level >10 ng/mL or reported smoking within the past 5 days; (2) “passive smoker” if they had a serum cotinine level of 0.05–10 ng/mL or reported the presence of smokers indoors without smoking themselves in the past 5 days; or (3) “non-smoker” if they had a serum cotinine level < 0.05 ng/mL or reported no indoor smokers and had not smoked in the past 5 days. In this study, subjects with fasting glucose levels ≥ 126 mg/dL, HbA1c levels ≥ 6.5%, or a self-reported diagnosis were defined as having diabetes. Hypertension was determined by a systolic blood pressure (SBP) ≥ 140 mmHg, diastolic blood pressure (DBP) ≥ 90 mmHg, or self-reported treatment for hypertension [33,34].

### 2.5. Statistical Analyses

A frequency (n) and percentage (%) were used to describe categorical variables, with chi-squared tests employed for group comparisons. Continuous variables are presented as means with standard deviations (mean ± SD) or medians with interquartile ranges (M, IQR), and Mann–Whitney U tests were used for comparisons. The normal distributions of all continuous variables were assessed using the Kolmogorov–Smirnov (KS) test. Due to the skewed distribution, concentrations of trace metals, serum ferritin, and hsCRP were natural logarithm (ln) transformed to improve normality when treated as continuous variables in statistical models. Spearman rank correlation was used to assess the correlations among the five trace metals.

#### 2.5.1. Logistic Regression Model

Logistic regression models were employed to assess the association between individual trace metals and anemia. The concentrations of these metals were categorized into four quartiles (Q1, Q2, Q3, and Q4) based on their distribution. Three logistic regression models were established to adjust to different covariates. In model Ⅰ, we adjusted for all covariates, including age, race, BMI, physical activity, family income, smoking status, drinking status, hypertension, and diabetes mellitus. Additionally, model Ⅱ was further adjusted for all other trace metals. Effect estimates were presented as odds ratios (ORs) along with their 95% confidence intervals (CIs) for anemia across the quartiles of trace metals (Q2, Q3, and Q4), with Q1 serving as the reference group.

#### 2.5.2. Quantile G-Computation (QGC) Regression Model

A QGC model was employed to evaluate both the combined and individual effects of trace metals on the risk of anemia. This method constructs a weighted index and assigns the corresponding weights of individual components in different directions, overcoming the orientational homogeneity limitation of weighted quantile sum (WQS) regression [35]. The model estimated the OR and its 95%CI for anemia associated with each quartile increase in the trace metal mixture using 1000 bootstrap samples. Metals with estimated weights greater than 0.05 were considered to contribute substantially to the QGC score.

#### 2.5.3. Bayesian Kernel Machine Regression (BKMR) Model

In considering possible nonlinear and nonadditive associations among trace metals, as well as additional supplementation for the QGC model, we employed the BKMR model [36]. Trace metal concentrations were ln-transformed and scaled to mitigate the impact of extreme values and differences in metrics before model fitting. In our study, BKMR was fitted using a probit regression model with component-wise variable selection. This model involved 10,000 iterations using the Markov chain Monte Carlo algorithm after adjusting for all covariates. BKMR was primarily utilized to explore and visualize three types of exposure–response functions: (1) the cumulative effects of mixtures on the risk of anemia, with simultaneous changes in all components of the mixture from their median levels; (2) univariate exposure–response curves of individual metals with all other metals fixed at their respective median levels; and (3) the single effect of individual metals for an interquartile range (IQR) increase while holding all other metals at their 25th, 50th, or 75th percentiles. BKMR also enabled estimation of the impact of individual metals on the risk of anemia through calculation of the posterior inclusion probability (PIP), using a significance threshold of 0.5 for determination.

#### 2.5.4. Sex-Stratified Analysis

Previous epidemiological evidence suggested that females exhibit higher susceptibility to anemia [37,38]. Given the potential sex-specific associations between trace metals and anemia, we further fitted the aforementioned models by sex to assess potential effect of sex.

#### 2.5.5. Mediation Analysis

A linear regression model was utilized to explore the associations between ln-transformed trace metals and iron status and inflammatory biomarkers, with coefficient β and its 95%CI representing the effect estimate. We then employed causal mediation analysis to investigate the potential mediating roles of serum ferritin, albumin, and hsCRP in the relationship between trace metals and anemia while utilizing nonparametric bootstrapping (*n* = 1000). This approach allowed us to derive several key indicators, including the average causal mediation effect (ACME), average direct effect (ADE), total effect (TE), and mediation proportion (%).

All statistical analyses in this study were conducted using R software (version 4.0.3) with the following packages: “stats” for logistic and linear regression, “qgcomp” for QGC analysis, “bkmr” for Bayesian kernel machine regression, and “mediation” for mediation analysis. All statistical tests were two-sided, and significance was determined with *p* < 0.05.

## 3. Results

### 3.1. Population Characteristics

Table 1 presents the characteristics and trace metal concentrations of the study participants. Among them, a total of 155 participants (12.17%) exhibited anemia, which was more frequently observed in individuals with hypertension, diabetes, and older ages (all *p* < 0.05). Significant differences in race, alcohol consumption, and smoking status were observed between anemic and non-anemic individuals (all *p* < 0.05). Compared with the non-anemic participants, those with anemia had lower levels of Se and Mn but higher levels of Co and Ni (all *p* < 0.05). Additionally, participants with anemia had significantly lower concentrations of serum ferritin and albumin, while their hsCRP levels were significantly higher compared with those for the participants without anemia (all *p* < 0.05). The correlations between the five trace metals were weak, with all Spearman correlation coefficients being below 0.3, indicating the absence of multicollinearity (Figure S2).

### 3.2. Trace Metals with Risk of Anemia in Logistic Regression Model

To evaluate the potential relationship between the quartiles of trace metals and the risk of anemia, we employed both univariate and multivariate logistic models. The results in Table 2 demonstrate a high degree of consistency across the three logistic models. Given that model Ⅱ accounted for more confounders, it was selected as the primary model for subsequent analyses. We observed an increased risk of anemia in the highest quartile of Ni and Co, while a decreased risk of anemia was observed for higher Se and Mn levels. Specifically, compared with the corresponding Q1, the risk of anemia increased 1.72 fold (95%CI: 1.14, 3.12) for Ni and 3.34 fold (95%CI: 1.96, 5.81) for Co in the highest quartiles (all *p* < 0.05). Mn showed a protective effect in Q3 with an OR of 0.54 (95%CI: 0.30, 0.96) (*p* < 0.05). For Se, the ORs of anemia were 0.55 (95%CI: 0.34, 0.88) in Q2, 0.31 (95%CI 0.18, 0.52) in Q3, and 0.25 (95%CI 0.14, 0.43) in Q4 compared with Q1 (all *p* < 0.05).

In sex-stratified analysis, the association between the Ni level and an increased risk of anemia was more pronounced in females (OR = 3.22, 95%CI: 1.31, 8.56) but was not significant in males. Similarly, higher OR values for Co were observed in females (OR = 4.12, 95%CI: 1.88, 9.54) compared with males (OR = 2.68, 95%CI: 1.16, 6.28) (all *p* < 0.05). The association between Se and anemia was consistent across sex-stratified subgroups. Conversely, the protective effect of Mn was more significant in males, with the risk of anemia reduced 0.12 fold (95%CI: 0.03, 0.36) in Q3 and 0.38 fold (95%CI: 0.14, 0.94) in Q4 compared with Q1 (all *p* < 0.05), while the effects were not significant in females (Tables S2 and S3). The differences in the associations between the sex-specified subgroups suggest potential sex-specific susceptibility.

### 3.3. Trace Metals with Risk of Anemia in QGC Model

**Figure 1:**
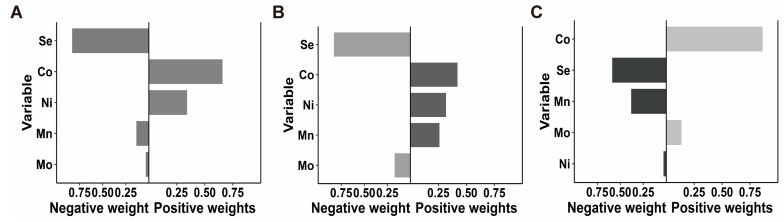
The QGC model weights of the trace metals for anemia in the overall population (**A**), females (**B**), and males (**C**). The model was adjusted for age, sex, race, BMI, physical activity, family income, smoking status, drinking status, hypertension, and diabetes mellitus. In the stratified analysis by sex, the full model was adjusted all the covariates without sex. Abbreviations: Se = selenium; Mn = manganese; Co = cobalt; Ni = nickel; Mo = molybdenum.

We utilized the QGC model to assess the combined effects of the five trace metals on anemia. The individual weights of each metal were used to present the importance of these metals (Table S4). As illustrated in Figure 1, no significant association was found between the trace metal mixture and the risk of anemia in the overall population. However, the stratified analysis by sex revealed a marginal association between the trace metals and an increased risk of anemia in females (OR = 1.44, 95%CI 0.96, 2.21) and a significantly association between the trace metals and a decreased risk of anemia in males (OR = 0.49, 95%CI 0.37, 0.92). Additionally, Co consistently presented the highest positive weight, whereas Se had the highest negative weight across the overall, male, and female populations.

### 3.4. Trace Metal Exposure with Risk of Anemia in BKMR Model

**Figure 2:**
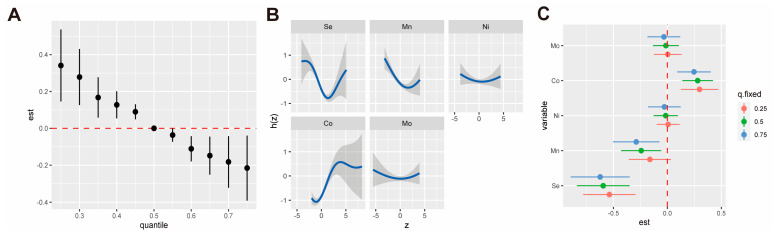
The associations between trace metals and anemia identified by the BKMR model in the overall population. (**A**) Overall associations of the trace metal mixture with risk of anemia at increasing percentiles compared to medians. (**B**) Univariate exposure-response function between individual metal with the risk of anemia with other metals fixed at the corresponding 50th percentiles. (**C**) Single-exposure effect of individual metals for an IQR increase on the risk of anemia when other metals are fixed at their 25th, 50th, or 75th percentiles. The model was adjusted for age, sex, race, BMI, physical activity, family income, smoking status, drinking status, hypertension, and diabetes mellitus. Abbreviations: Se = selenium; Mn = manganese; Co = cobalt; Ni = nickel; Mo = molybdenum.

We used the BKMR model to further assess both the combined and individual effects of trace metals on anemia, with the PIP value used to estimate the importance of individual metals. Figure 2A illustrated the combined effects of these metals on anemia relative to their 50th percentile, as determined by the BKMR model. The results indicate that higher levels of trace metals were significantly associated with a decreased risk of anemia in the overall population. Among these metals, Se (PIP = 1.000), Mn (PIP = 0.957), and Co (PIP = 1.000) exhibited the highest PIPs, suggesting these metals may be the most significant contributors to anemia (Table S5). In sex-stratified analysis (Figures S3 and S4), we found significant associations between the trace metals and a decreased risk of anemia in both females and males. However, the combined effect of a trace metal mixture was more pronounced in males (β = −0.43, 95%CI −0.78, −0.10) than in females (β = −0.06, 95%CI −0.21, 0.07), according to the estimate effect value of the 75th percentile compared with the corresponding medians.

The univariate exposure–response curve for individual metals is shown in Figure 2B, with other metals fixed at their median levels. The individual effect of single metals for an IQR increase is demonstrated in Figure 2C, where other metals were fixed to the 25th, 50th, and 75th percentiles. The analysis revealed that the Mn and Se levels were associated with a decreased risk of anemia, while the Co levels were associated with an increased risk of anemia. Additionally, we identified a “U-shaped” exposure–response curve for Se. In the sex-stratified analysis, the protective effect of Se was consistent in both females and males. However, the effect of Mn was not significant in females, and the association between Co and anemia was not significant in the male population (Figures S3 and S4).

### 3.5. The Mediating Effect of Iron Status and Inflammation

As shown in Table S6, each unit increase in Ni, Co, and Mn was significantly associated with a decrease in the ln-transformed serum ferritin levels of −0.19 (95%CI: −0.26, −0.11), −0.45 (95%CI: −0.52, −0.37), and −0.57 (95%CI: −0.72, −0.45), respectively. Conversely, each unit increase in Se was associated with an increase in the ln-transformed ferritin levels by 0.88 (95%CI: 0.55, 1.22). Additionally, an association between Co and decreased serum albumin was observed (β = −0.83, 95%CI: −1.10, −0.57, *p* < 0.05), while Se demonstrated a positive association with albumin (β = 4.28, 95%CI: 3.14, 5.43). Furthermore, Ni was associated with increased ln-transformed hsCRP levels (β = 0.10, 95%CI: 0.01, 0.19), whereas Se showed a negative association (β = −0.59, 95%CI: −0.99, −0.18).

As shown in Table S7, the ln-transformed serum ferritin and albumin levels were associated with a decreased risk of anemia, with ORs of 0.94 (95%CI: 0.92, 0.96) and 0.98 (95%CI: 0.98, 0.99), respectively. In contrast, the ln-hsCRP levels were associated with an increased risk of anemia, with an OR of 1.03 (95%CI: 1.01, 1.04).

We further conducted causal mediation analysis to investigate the potential mediating effects of serum ferritin, albumin, and hsCRP on the associations between trace metals and anemia. As presented in Table 3, we found that serum ferritin significantly mediated the associations of Ni, Co, and Se with anemia, with the mediation proportions being 58.18%, 46.46%, and 14.72%, respectively (all *p* for mediation < 0.05). Furthermore, albumin mediated the associations between Ni, Co, and Se and anemia, accounting for 15.83%, 20.75%, and 23.26% of the effects, respectively (all *p* for mediation < 0.05). Similarly, hsCRP also played mediating roles in the association between Ni and Se and anemia, accounting for 10.19% and 13.30% of the effects, respectively.

## 4. Discussion

Using the NHANES 2017–2020 data from the US adult population, we investigated the individual and combined effects of trace metals (Ni, Co, Mn, Se, and Mo) on anemia and further assessed the mediating role of the iron status and inflammation. Our results indicate that the trace metal mixture was associated with a decreased risk of anemia, and the association was primarily driven by Se and Co. Further sex-stratified analysis revealed that the protective effect of the trace metal mixture on anemia was more pronounced in males. Finally, mediation analysis showed that the iron status and inflammation played significant roles in these associations.

When analyzing the combined effect of the trace metal mixture, we observed that a simultaneous increase in all components of the mixture was associated with a lower risk of anemia. To our knowledge, the association between the trace metal mixture and anemia has been rarely explored. Schildroth et al. investigated associations between metal mixtures of Pb, Cd, Mn, and Se and various anemia-related parameters in adolescents from the NHANES, and they found that the metal mixture was positively associated with hemoglobin and mean cellular hemoglobin levels, which are indicators of anemia [23]. Consistent with our findings, the protective effect of the metal mixture was primarily driven by Se, which may be attributed to its antioxidant properties [39]. Given the high dietary intake, the average level of blood Se in the US general population is higher than those in other countries [40,41,42]. Consistent with previous biomonitoring data reported by the CDC [26], the median of blood Se was 184.69 µg/L in our study, significantly below the established toxic threshold of 1000 µg/L [43]. Similarly, several other studies reported individual Se exposure and decreased risk of anemia in various populations [44,45,46]. Additionally, the association between Mn exposure and a decreased risk of anemia was also reported. This finding may be attributed to Mn’s role in enzyme activation and its involvement in the formation of antioxidants, which help protect red blood cells from oxidative stress [22,47].

Qiao et al. investigated the relationship between a mixture of 11 urinary metals and anemia in an elderly population [13]. Although their study included trace metals like Co and Se, it mainly focused on toxic metals such as Te, As, and Pb, reporting the association between the metal mixture and increased anemia risk. Consistent with our findings, Co was identified as a primary component contributing to adverse effects in Qiao’s study. Similarly, Fort et al. reported an association between individual Co exposure and lower hemoglobin levels in pregnant women [21]. Despite its role as a component of vitamin B12, Co can be acutely cytotoxic at high concentrations, increasing pro-inflammatory cytokine secretion and oxidative stress, which may explain the adverse effect of Co on anemia [48,49]. Although different biological samples were used to measure Ni and Mo, the internal consistency in comparing each metal between anemic and non-anemic populations ensured the reliability of the estimations regarding their associations with anemia risk. While no previous study assessed the effect of Ni on anemia within metal mixtures, several studies observed significantly higher concentrations of Ni in anemic populations than non-anemic populations, suggesting its potential adverse effects [50,51]. The underlying mechanisms may involve not only the competitive inhibition of iron absorption via DMT1 but also Ni-induced inflammation and oxidative stress [19,52]. No significant association was found between Mo and anemia. However, a previous animal experiment showed the relationships between Mo exposure and reduced hematological parameters, including the RBC count and hemoglobin concentration [53]. Thus, further investigation into Mo’s association with anemia is warranted.

Iron deficiency and inflammation are key mechanisms contributing to the development of anemia. Iron is essential for hemoglobin synthesis, with most of the body’s iron recycled for erythropoiesis. Only 1 mg of iron is lost daily, which is replenished through absorption from duodenal enterocytes [18]. Disruptions in iron metabolism due to inadequate intake, poor absorption, or chronic blood loss overwhelm the body’s compensatory mechanisms, resulting in anemia [54,55]. Serum ferritin serves as a marker of long-term iron stores and has been widely utilized to evaluate one’s iron status [56,57,58]. As expected, significant associations between anemia risk and serum ferritin were observed, confirming the crucial roles of the iron status in anemia development.

Inflammation can induce anemia through various pathways. Pro-inflammatory cytokines, such as interleukin-6 (IL-6), upregulate hepcidin transcription via the transcription 3 (STAT3) pathway [59,60,61]. Elevated hepcidin levels decrease intestinal iron absorption and sequester iron in macrophages, reducing its availability for erythropoiesis [62]. Additionally, inflammation can directly inhibit erythropoiesis and increase phagocytosis [17,63,64]. In this study, serum albumin and hsCRP were used as inflammatory biomarkers. CRP is a well-established marker of acute inflammation [65,66], while albumin reflects chronic inflammation levels due to its longer half-life and lower variability [67,68,69,70,71,72]. Previous studies linked hypoalbuminemia with anemia across various populations, suggesting that decreased albumin levels may be associated with inflammatory processes, contributing to anemia [73,74,75]. Our study similarly observed relationships between low albumin levels and higher hsCRP levels with an increased risk of anemia, further supporting the significant role of inflammation in anemia development.

Accumulating evidence supports associations between trace metals and both iron status and inflammation. For instance, Zhou et al. reported elevated serum iron levels in relation to higher Se exposure [46]. This relationship is likely due to Se’s ability to modulate hepcidin levels, promoting efficient iron uptake and utilization [76]. As a key component of antioxidant selenoproteins, Se regulates inflammation by suppressing oxidative stress and reducing pro-inflammatory cytokines such as IL-6 and TNF-α [77,78]. Consistent with our findings, Junqué et al. reported a negative relationship between urinary Co levels and serum ferritin, with a similar adverse effect observed for Ni in pregnant women [51,79]. The detrimental impact of Ni and Co on iron status largely due to their competition with iron for absorption in enterocytes, particularly via the divalent metal transporter-1 (DMT1) pathway [19,80].

Furthermore, Co and Ni can exacerbate inflammatory responses at higher concentrations. It has been demonstrated that Co can increase pro-inflammatory cytokine secretion, such as IL-8 and IL-6, through Toll-like receptor 4 activation [48]. Co also inhibits mitochondrial function, leading to oxidative stress [49]. Similarly, Ni enhances inflammatory responses by promoting oxidative stress [52]. Given the shared pathogenesis, it is reasonable to investigate whether the iron status and inflammation participate in the relationships between trace metals and anemia risk. In this study, we found that serum ferritin, albumin, and hsCRP were involved in the association between Ni, Co, and Se and anemia, accounting for the proportions ranging from 10.29% to 58.18% when using mediation analysis. Further studies are warranted to confirm these underlying mechanisms.

In the sex-stratified analysis, females exhibited a higher susceptibility to anemia, consistent with previous studies [23,81]. This disparity may be attributed to hormonal differences, with testosterone promoting erythropoiesis by stimulating erythropoietin production and suppressing hepcidin, thus enhancing iron utilization [82]. Conversely, elevated estrogen levels in females can increase hepcidin expression, leading to reduced iron absorption and utilization [83,84]. Trace metals such as Ni, Co, and Mn are involved in hormone regulation and have been linked to variations in serum testosterone and estrogen levels [85,86,87]. Luo et al. also reported that testosterone mediated the relationships between metal exposure and hemoglobin levels [88]. Additionally, other factors, including metal accumulation, genetic predispositions, and metabolic conditions, may contribute to these observed differences [89]. However, further research is needed to better understand the sex-specific impacts of these trace metals on anemia risk.

As the most recent study exploring the associations between trace metal exposure and anemia risk, our study presents several notable strengths. Firstly, our study utilized a nationally representative sample of the US population, enhancing the generalizability of our findings. Secondly, we employed various statistical methods and adjusted for potential confounding variables to bolster the robustness and reliability of our results. Additionally, we examined possible mechanisms underlying the relationships between trace metal exposure and anemia risk, finding that the iron status and inflammation significantly mediated these associations, providing a valuable foundation for future mechanistic research.

Despite these strengths, our study has limitations. Firstly, the cross-sectional design precludes the establishment of causal relationships between trace metal exposure, iron status, inflammation, and anemia risk. Secondly, comparing metal content across different biological matrices may introduce bias in result interpretation. Furthermore, our analysis was limited to five trace metals due to undetectable or below-detection-limit levels of other metals in the NHANES dataset for most participants. Lastly, we did not fully investigate the potential interaction effects among metal mixtures, highlighting the need for further research in this area.

## 5. Conclusions

In conclusion, our study found that trace metal mixtures were significantly associated with a lower risk of anemia, with Se and Co identified as the main contributors. The protective effects were less pronounced in females, indicating a higher susceptibility. Moreover, the iron status and inflammation acted as mediators linking trace metal exposure to anemia risk. These findings highlight the critical role of trace metals in anemia development. More prospective cohort and mechanism studies are warranted to validate the potential role and biological mechanism of trace metals in anemia regulation.

## Figures and Tables

**Table 1:** Characteristics of participants in US adults (NHANES 2017–2020).

Characteristics	Total	Anemic	Non-Anemic	*p* Value
	(N = 1274)	(N = 155)	(N = 1119)	
Age (year), mean ± SD	59.87 ± 11.45	62.54 ± 12.87	59.50 ± 11.19	0.006
Gender, *n* (%)				0.078
Male	672 (52.74)	71 (45.81)	601 (53.71)	
Female	602 (47.25)	84 (54.19)	518 (46.29)	
Race, *n* (%)				<0.001
Mexican American	123 (9.65%)	14 (9.03%)	109 (9.74%)	
Other Hispanic	118 (9.26%)	6 (3.87%)	112 (10.01%)	
Non-Hispanic White	535 (41.99%)	48 (30.97%)	487 (43.52%)	
Non-Hispanic Black	328 (25.75%)	78 (50.32%)	250 (22.34%)	
Other Race	170 (13.34%)	9 (5.81%)	161 (14.39%)	
BMI (kg/m^2^), mean ± SD	30.57 ± 7.16	31.58 ± 8.56	30.43 ± 6.93	0.109
Smoking, *n* (%)				0.007
Non-smoker	697 (54.71%)	90 (58.06%)	607 (54.24%)	
Passive smoker	276 (21.66%)	43 (27.74%)	233 (20.82%)	
Active smoker	301 (23.63%)	22 (14.19%)	279 (24.93%)	
Drinking, *n* (%)				0.008
Non-drinker	329 (25.82%)	51 (32.90%)	278 (24.84%)	
Moderate drinker	584 (45.84%)	75 (48.39%)	509 (45.48%)	
Heavy drinker	361 (28.34%)	29 (18.71%)	332 (29.67%)	
PIR, *n* (%)				0.548
<1	189 (14.84%)	20 (12.90%)	169 (15.10%)	
≥1	1085 (85.16%)	135 (87.10%)	950 (84.90%	
Hypertension, *n* (%)				<0.001
Yes	770 (60.44%)	116 (74.84%)	654 (58.44%)	
No	504 (39.56%)	39 (25.16%)	465 (41.55%)	
Diabetes, *n* (%)				<0.001
Yes	354 (27.79%)	66 (42.58%)	288 (25.73%)	
No	920 (72.21%)	89 (57.42%)	831 (74.26%)	
Physical activity *n* (%)				0.497
Active	579 (45.45%)	66 (42.58%)	513 (45.84%)	
Inactive	695 (54.55%)	89 (57.42%)	606 (45.84%)	
Ferritin (μg/L), median (IQR)	124.00 (64.03, 225.00)	81.60 (19.25, 173.00)	131.00 (69.95, 229.50)	<0.001
Albumin (g/L), median (IQR)	40.32 ± 3.16	38.26 ± 2.98	40.60 ± 3.07	<0.001
hsCRP(mg/L), median (IQR)	2.17 (0.98, 4.64)	2.94 (1.14, 6.35)	2.14 (0.97, 4.37)	0.002
Trace metal concentrations, median (IQR)				
Se (μg/L)	184.69 (169.08, 201.55)	171.66 (168.16, 189.95)	185.92 (171.00, 202.65)	<0.001
Mn (μg/L)	8.98 (7.10, 11.02)	8.43 (6.61, 10.89)	9.04 (7.20, 11.02)	0.015
Co (μg/L)	0.14 (0.11, 0.18)	0.16 (0.12, 0.27)	0.14 (0.10, 0.17)	<0.001
Ni (μg/g cre)	1.15 (0.76, 1.83)	1.33 (0.86, 2.25)	1.12 (0.74, 1.79)	0.004
Mo (μg/g cre)	33.87 (21.70, 50.14)	30.80 (21.42, 49.47)	33.93 (21.83, 50.36)	0.405

Differences in continuous variables between anemic and non-anemic individuals were assessed using Student’s *t*-test or Mann–Whitney U test. Differences in categorical variables were assessed by chi-squared tests. Abbreviations: BMI = body mass index; PIR = family poverty income ratio; Se = selenium; Mn = manganese; Co = cobalt; Ni = nickel; Mo = molybdenum.

**Table 2:** Multivariate logistic regression analysis for risk of anemia in overall population associated with quartiles of trace metals.

Metals		Crude Model	*p* Value	Model Ⅰ	*p* Value	Model Ⅱ	*p* Value
		Crude OR (95%CI)		Adjusted OR (95%CI)		Adjusted OR (95%CI)	
Ni	Q1	Ref		Ref		Ref	
	Q2	1.11 (0.66, 1.88)	0.691	1.14 (0.65, 1.99)	0.634	1.11 (0.62, 1.97)	0.742
	Q3	1.27 (0.77, 2.12)	0.352	1.46 (0.84, 2.57)	0.183	1.18 (0.65, 2.14)	0.595
	Q4	2.01 (1.26, 3.26)	0.004	2.34 (1.37, 4.06)	0.002	1.72 (1.14, 3.12)	0.006
Co	Q1	Ref		Ref		Ref	
	Q2	1.12 (0.68, 1.84)	0.658	1.23 (0.73, 2.09)	0.435	1.55 (0.90, 2.69)	0.112
	Q3	0.94 (0.54, 1.60)	0.814	1.06 (0.59, 1.86)	0.848	1.40 (0.76, 2.55)	0.272
	Q4	2.49 (1.59, 3.86)	<0.001	2.97 (1.82, 4.92)	<0.001	3.34 (1.96, 5.81)	<0.001
Mn	Q1	Ref		Ref		Ref	
	Q2	0.93 (0.59, 1.44)	0.734	1.01 (0.63, 1.63)	0.953	1.01 (0.61, 1.68)	0.970
	Q3	0.52 (0.31, 0.85)	0.011	0.62 (0.36, 1.06)	0.083	0.54 (0.30, 0.96)	0.036
	Q4	0.78 (0.49, 1.24)	0.295	1.18 (0.70, 2.00)	0.537	0.93 (0.52, 1.64)	0.798
Se	Q1	Ref		Ref		Ref	
	Q2	0.51 (0.33, 0.77)	0.002	0.53 (0.34, 0.84)	0.007	0.55 (0.34, 0.88)	0.013
	Q3	0.31 (0.19, 0.50)	<0.001	0.31 (0.18, 0.51)	<0.001	0.31 (0.18, 0.52)	<0.001
	Q4	0.27 (0.16, 0.44)	<0.001	0.27 (0.15, 0.45)	<0.001	0.25 (0.14, 0.43)	<0.001
Mo	Q1	Ref		Ref		Ref	
	Q2	0.98 (0.61, 1.56)	0.917	1.22 (0.74, 2.00)	0.440	1.07 (0.64, 1.81)	0.772
	Q3	0.87 (0.54, 1.39)	0.553	1.01 (0.60, 1.68)	0.978	0.87 (0.51, 1.50)	0.630
	Q4	0.92 (0.57, 1.47)	0.718	1.06 (0.63, 1.77)	0.835	1.01 (0.58, 1.75)	0.987

The crude model did not adjust for any covariates. Model Ⅰ adjusted for all covariates, including age, sex, race, BMI, physical activity, family income, smoking status, drinking status, hypertension, and diabetes mellitus. Model Ⅱ further adjusted for all other trace metals. Abbreviations: Se = selenium; Mn = manganese; Co = cobalt; Ni = nickel; Mo = molybdenum; 95%CI = 95% confidence interval.

**Table 3:** The mediation effect of the iron status and inflammation on the association of trace metals and the risk of anemia.

Mediators	Metals	TE	ACME	Mediated Proportion (%)	*p* Value
Ln-ferritin	Ni	3.81 × 10^−2^ (1.15 × 10^−2^, 0.06)	2.22 × 10^−2^ (1.45 × 10^−2^, 0.03)	58.18%	0.004
	Co	7.54 × 10^−2^ (4.85 × 10^−2^, 0.11)	3.55 × 10^−2^ (2.49 × 10^−2^, 0.05)	46.46%	<0.001
	Mn	1.85 × 10^−2^ (−3.44 × 10^−2^, 0.07)	6.30 × 10^−2^ (4.57 × 10^−2^, 0.08)		
	Se	−3.59 × 10^−1^ (−4.92 × 10^−1^, −0.23)	−5.32 × 10^−2^ (−8.64 × 10^−2^, −0.03)	14.72%	<0.001
	Mo	−2.31 × 10^−3^ (−3.81 × 10^−3^, 0.01)	7.99 × 10^−4^ (−2.80 × 10^−2^, 0.03)		
Albumin	Ni	3.69 × 10^−2^ (9.19 × 10^−3^, 0.06)	6.21 × 10^−3^ (1.40 × 10^−4^, 0.01)	15.83%	0.042
	Co	7.60 × 10^−2^ (4.78 × 10^−2^, 0.10)	1.58 × 10^−2^ (9.73 × 10^−3^, 0.02)	20.75%	<0.001
	Mn	1.96 × 10^−2^ (−4.05 × 10^−2^, 0.08)	−6.29 × 10^−3^ (−4.47 × 10^−2^, 0.08)		
	Se	−3.61 × 10^−1^ (−0.48, −0.23)	−8.42 × 10^−2^ (−1.24 × 10^−1^, −0.05)	23.26%	<0.001
	Mo	−2.61 × 10^−5^ (−2.62 × 10^−2^, 0.03)	2.18 × 10^−3^ (−3.26 × 10^−3^, 0.01)		
Ln-hsCRP	Ni	3.66 × 10^−2^ (8.87 × 10^−3^, 0.06)	3.87 × 10^−3^ (0.00, 7.99 × 10^−3^)	10.19%	0.004
	Co	7.67 × 10^−2^ (4.80 × 10^−2^, 0.11)	−1.11 × 10^−3^ (−3.96 × 10^−3^, 0.01)		
	Mn	1.83 × 10^−2^ (−3.94 × 10^−2^, 0.07)	−4.33 × 10^−3^ (−1.08 × 10^−2^, 0.01)		
	Se	−3.62 × 10^−1^ (−0.48, −0.25)	−4.87 × 10^−2^ (−7.67 × 10^−2^, −0.02)	13.30%	<0.001
	Mo	−3.62 × 10^−4^ (−2.66 × 10^−2^, 0.03)	−5.32 × 10^−3^ (9.80 × 10^−3^, 0.01)		

Trace metals were ln-transformed and individually included in generalized linear regression models, with adjustments for age, sex, race, BMI, physical activity, family income, smoking status, drinking status, hypertension, and diabetes mellitus. Abbreviations: ACME = average causal mediation effects; TE = total effect; Se = selenium; Mn = manganese; Co = cobalt; Ni = nickel; Mo = molybdenum.

## Data Availability

The data analyzed in this study is openly accessible via the NHANES website (https://wwwn.cdc.gov/nchs/nhanes/Default.aspx, accessed on 15 May 2024).

## Supplementary Materials

The following supporting information can be downloaded at https://www.mdpi.com/article/10.3390/nu16193424/s1. Table S1: Detection rates of six trace metals in NHANES 2017–2020. Table S2: Multivariate logistic regression analysis for risk of anemia in females according to quartiles of trace metals. Table S3: Multivariate logistic regression analysis for risk of anemia in males according to quartiles of trace metals. Table S4: Associations of QGC indexes of trace metals with risk of anemia. Table S5: PIPs obtained using the BKMR model for each trace metal with anemia. Table S6: Association between trace metals and iron status and inflammation. Table S7: The effects of the iron status and inflammation biomarkers on the risk of anemia. Figure S1: Flowchart of participants included in this study. Figure S2: Spearman correlations among the five trace metals. Figure S3: The associations between trace metals with anemia identified by the BKMR model in females. Figure S4: The associations between trace metals and anemia identified by the BKMR model in males.
