# Supplementary material for: Exploring Associations and Mediating Factors between Multiple Trace Metals with Anemia in US Adults: Insight from NHANES 2017–2020
Source: Nutrients. 2024 Oct 9;16(19):3424. doi: 10.3390/nu16193424 (PMC11478990; doi:10.3390/nu16193424)
Supplement: Supplementary file 1 [file nutrients-16-03424-s001.zip › nutrients-3212374-supplementary.pdf]

Supplementary Material

Table S1 Distributions of the metal concentration in NHANES 2017-2020

Table S2 Multivariate logistic regression analysis for risk of anemia in females associated with quartiles of trace metals.

Table S3 Multivariate logistic regression analysis for risk of anemia in males associated with quartiles of trace metals.

Table S4 Associations of QGC index of trace metals with risk of anemia.

Table S5 PIPs obtained using the BKMR model for each trace metals with anemia.

Table S6 Association between trace metals and iron status and inflammation.

Table S7 The effects of iron status and inflammation biomarkers on the risk of anemia.

Figure S1 Flowchart of participants included in this study.

Figure S2 Spearman correlations among the five trace metals.

Figure S3 The associations between trace metals with anemia identified by the BKMR model in females.

Figure S4 The associations between trace metals with anemia identified by the BKMR model in males.

Table S1 Distributions of the metal concentration in NHANES 2017-2020

Metals	Biological specimens	Concentration			Reference value	Citation source	Detection rate (%)
		25th	50th	75th			
Pb[a]	blood	0.44	0.73	1.24	< 5ug/dL	CDC	99.96%
Cd[b]	blood	0.11	0.21	0.40	<1ug/L	Literature	80.31%
Hg[b]	blood	0.20	0.46	1.06	<5ug/L	Literature	68.57%
Mn[b]	blood	7.60	9.48	11.79	5~18.8ug/L	Literature	100.00%
Se[b]	blood	164.00	179.28	196.33	146.8~247.35ug/L	Literature	100.00%
Co[b]	blood	0.11	0.14	0.18	<0.48ug/L	CDC	95.56%
Cr[b]	blood	0.29	0.29	0.29	<0.86ug/L	CDC	17.90%
Ba[b]	urine	0.50	1.07	2.13	<6.13ug/L	CDC	98.89%
Cd[b]	urine	0.04	0.12	0.29	<0.90ug/L	CDC	70.40%
Pb[b]	urine	0.16	0.29	0.52	<1.31ug/L	CDC	99.18%
Cs[b]	urine	2.74	4.65	6.83	<13.7ug/L	CDC	100.00%
Sb[b]	urine	0.03	0.05	0.09	0.24ug/L	CDC	82.94%
Sn[b]	urine	0.26	0.57	1.38	<4.57ug/L	CDC	82.09%
Tl[b]	urine	0.11	0.18	0.28	<0.52ug/L	CDC	82.09%
W[b]	urine	0.04	0.08	0.16	<0.40ug/L	CDC	90.66%
Mo[b]	urine	22.62	44.07	76.90	<153ug/L	CDC	100.00%
Ni[b]	urine	0.71	1.29	2.22	<4.7ug/L	CDC	93.39%
As[b]	urine	3.07	5.90	12.31	<70.5ug/L	CDC	99.37%

[a] Measurement unit: µg/dL; [b] Measurement unit: µg/L

Abbreviations: Pb, Lead; Cd, Cadmium; Hg, Mercury; Mn, Manganese; Se, Selenium; Co, Cobalt; Cr, Chromium; Ba, Barium; Cs, Cesium; Sb, Antimony; Sn, Tin; Tl, Thallium; W, Tungsten; Mo, Molybde num; Ni, Nickel; As, Arsenic.

Table S2 Multivariate logistic regression analysis for risk of anemia in females associated with quartiles of trace metals.

Metals		Crude Model	*P* value	Model Ⅰ	*P* value	Model Ⅱ	*P* value
		Crude OR (95%CI)		Adjusted OR (95%CI)		Adjusted OR (95%CI)	
Ni	Q1	Ref		Ref		Ref	
	Q2	1.58(0.70, 3.82)	0.281	1.81(0.76, 4.56)	0.187	1.92(0.77, 5.05)	0.171
	Q3	1.59(0.72, 3.79)	0.270	2.18(0.93, 5.50)	0.083	1.80(0.71, 4.86)	0.227
	Q4	2.44(1.17, 5.61)	0.024	3.84(1.71, 9.44)	0.002	3.22(1.31, 8.56)	0.014
Co	Q1	Ref		Ref		Ref	
	Q2	1.19(0.56, 2.56)	0.642	1.39(0.62, 3.16)	0.420	1.68(0.72, 3.97)	0.229
	Q3	0.98(0.45, 2.12)	0.950	1.16(0.51, 2.67)	0.720	1.62(0.67, 3.97)	0.288
	Q4	2.40(1.27, 4.78)	0.009	4.02(1.96, 8.67)	<0.001	4.12(1.88, 9.54)	<0.001
Mn	Q1	Ref		Ref		Ref	
	Q2	1.75(0.81, 4.02)	0.169	1.79(0.79, 4.30)	0.177	1.77(0.74, 4.45)	0.210
	Q3	1.51(0.70, 3.46)	0.308	1.78(0.78, 4.30)	0.181	1.63(0.69, 4.07)	0.276
	Q4	1.79(0.86, 4.00)	0.133	2.91(1.26, 7.17)	0.015	2.16(0.88, 5.60)	0.101
Se	Q1	Ref		Ref		Ref	
	Q2	0.51(0.28, 0.90)	0.023	0.55(0.29, 1.01)	0.057	0.52(0.26, 1.01)	0.054
	Q3	0.37(0.19, 0.69)	0.003	0.35(0.17, 0.68)	0.003	0.34(0.16, 0.69)	0.004
	Q4	0.27(0.13, 0.54)	<0.001	0.27(0.12, 0.56)	<0.001	0.21(0.09, 0.47)	<0.001
Mo	Q1	Ref		Ref		Ref	
	Q2	1.36(0.71, 2.62)	0.350	1.52(0.76, 3.08)	0.236	1.18(0.56, 2.53)	0.658
	Q3	0.98(0.51, 1.90)	0.961	1.26(0.63, 2.60)	0.497	0.87(0.41, 1.88)	0.729
	Q4	0.83(0.42, 1.65)	0.602	1.14(0.54, 2.43)	0.724	0.95(0.43, 2.12)	0.903

The crude model did not adjust for any covariates. Model I adjusted for all covariates, including age, race, BMI, physical activity, family income, smoking status, drinking status, hypertension and diabetes mellitus. Model II further adjusted for all other trace metals.

Abbreviations: Se, selenium; Mn, manganese; Co, cobalt; Ni, nickel; Mo, molybdenum; 95% CI: 95% confidence interval.

Table S3 Multivariate logistic regression analysis for risk of anemia in males associated with quartiles of trace metals.

Metals		Crude Model	*P* value	Model I	*P* value	Model II	*P* value
		Crude OR(95%CI)		Adjusted OR (95%CI)		Adjusted OR (95%CI)	
Ni	Q1	Ref		Ref		Ref	
	Q2	0.81(0.39,1.64)	0.570	0.70(0.31,1.51)	0.367	0.62(0.26, 1.42)	0.261
	Q3	1.06(0.53,2.09)	0.872	0.81(0.36,1.79)	0.602	0.60(0.24, 1.47)	0.269
	Q4	1.66(0.86,3.20)	0.131	1.27(0.58,2.82)	0.555	0.84(0.34, 2.03)	0.695
Co	Q1	Ref		Ref		Ref	
	Q2	1.04(0.53,2.04)	0.899	0.90(0.43,1.86)	0.776	1.46(0.67, 3.18)	0.339
	Q3	0.85(0.38,1.80)	0.683	0.71(0.30,1.64)	0.433	1.00(0.39, 2.49)	0.997
	Q4	2.43(1.26,4.70)	0.008	1.56(0.74,3.29)	0.238	2.68(1.16, 6.28)	0.021
Mn	Q1	Ref		Ref		Ref	
	Q2	0.68(0.38,1.19)	0.184	0.75(0.39,1.44)	0.402	0.73(0.37, 1.44)	0.370
	Q3	0.13(0.04,0.32)	<0.001	0.17(0.05,0.46)	0.001	0.12(0.03, 0.36)	<0.001
	Q4	0.32(0.13,0.67)	0.005	0.46(0.18,1.06)	0.083	0.38(0.14, 0.94)	0.044
Se	Q1	Ref		Ref		Ref	
	Q2	0.50(0.26,0.92)	0.030	0.54(0.26,1.09)	0.090	0.58(0.27, 1.20)	0.148
	Q3	0.25(0.11,0.51)	<0.001	0.28(0.12,0.62)	0.002	0.30(0.12, 0.68)	0.005
	Q4	0.27(0.12,0.53)	<0.001	0.29(0.13,0.63)	0.002	0.26(0.11, 0.60)	0.002
Mo	Q1	Ref		Ref		Ref	
	Q2	0.69(0.34,1.37)	0.295	1.03(0.48,2.19)	0.418	0.93(0.41, 2.10)	0.869
	Q3	0.72(0.35,1.46)	0.365	0.86(0.38,1.89)	0.963	0.86(0.36, 1.99)	0.727
	Q4	1.00(0.52,1.92)	0.997	1.14(0.54,2.40)	0.855	1.42(0.63, 3.23)	0.400

The crude model did not adjust for any covariates. Model Ⅰ adjusted for all covariates, including age, race, BMI, physical activity, family income, smoking status, drinking status, hypertension and diabetes mellitus. Model Ⅱ further adjusted for all other trace metals.

Abbreviations: Se, selenium; Mn, manganese; Co, cobalt; Ni, nickel; Mo, molybdenum; 95% CI: 95% confidence interval.

Table S4 Weights of each metal and overall effect estimate for trace metals for risk of anemia

Trace metals	Overall population	Female	Male
Ni	0.340	0.310	0.025
Co	0.660	0.433	0.873
Mn	-0.137	0.257	-0.407
Se	-0.833	-0.831	-0.593
Mo	-0.030	-0.169	0.132
Metal mixture OR(95%CI)	0.97(0.96,1.03)	1.44(0.96,2.21)	0.49(0.37,0.92)

Data are presented as the weights fo individual metals and estimated OR (95%CI) of metal mixture using QGC model, representing the single effect weight and combined effect for each quartile increase in the trace metal mixture on the risk of anemia. The model was adjusted for age, sex, race, BMI, physical activity, family income, smoking status, drinking status, hypertension and diabetes mellitus. In the stratified analysis by sex, the model adjusted all covariates without sex.

Table S5 PIPs obtained using the BKMR model for each trace metals with anemia.

Trace metals	Overall population	Female	Male
Se	1.000	1.000	1.000
Mn	0.957	0.705	0.897
Ni	0.435	0.613	0.433
Co	1.000	0.997	0.486
Mo	0.496	0.786	0.308

Abbreviations: PIP, posterior inclusion probability; BKMR, Bayesian kernel machine regression; Se, selenium; Mn, manganese; Co, cobalt; Ni, nickel; Mo, molybdenum

Table S6 Association between trace metals and iron status and inflammation.

Iron status and inflammation biomarkers	Essential metals	Crude model		Model I		Model II	
		β (95% CI)	*P* value	β (95% CI)	*P* value	β (95% CI)	*P* value
Ln-ferritin	Ni	-0.34(-0.42, -0.26)	<0.001	-0.30(-0.38, -0.22)	<0.001	-0.19(-0.26, -0.11)	<0.001
	Co	-0.59(-0.67, -0.51)	<0.001	-0.53(-0.61, -0.46)	<0.001	-0.45(-0.52, -0.37)	<0.001
	Mn	-0.91(-1.06, -0.75)	<0.001	-0.78(-0.94, -0.63)	<0.001	-0.57(-0.72, -0.45)	<0.001
	Se	0.75(0.36, 1.14)	<0.001	0.75(0.38, 1.11)	<0.001	0.88(0.55, 1.22)	<0.001
	Mo	-0.05(-0.13, 0.03)	0.236	-0.03(-0.11,0.05)	0.471	0.01(-0.06, 0.08)	0.781
Albumin	Ni	-0.21(-0.46, 0.05)	0.109	-0.27(-0.53, -0.02)	0.038	-0.09(-0.35, 0.16)	0.531
	Co	-0.78(-2.05, -0.52)	<0.001	-0.74(-0.99, -0.48)	<0.001	-0.83(-1.10, -0.57)	<0.001
	Mn	-0.15(-0.65, 0.36)	0.573	0.05(-0.46,0.55)	0.860	0.39(-0.12, 0.89)	0.123
	Se	4.85(3.63, 6.07)	<0.001	4.06(2.90, 5.21)	<0.001	4.28(3.14, 5.43)	<0.001
	Mo	0.15(-0.10, 0.41)	0.234	0.08(-0.16, 0.33)	0.504	0.06(-0.17, 0.31)	0.573
Ln-hsCRP	Ni	0.17(0.08, 0.26)	<0.001	0.13(0.05, 0.22)	0.003	0.10(0.01,0.19)	0.025
	Co	-0.07(-0.16, 0.03)	0.194	-0.04(-0.13, 0.05)	0.351	0.01(-0.09, 0.10)	0.905
	Mn	0.01(-0.17, 0.20)	0.889	-0.17(-0.34, 0.01)	0.066	-0.14(-0.32, 0.04)	0.130
	Se	-0.82(-1.27, -0.37)	<0.001	-0.60(-1.01, -0.19)	0.004	-0.59(-0.99, -0.18)	0.005
	Mo	-0.22(-0.31, -0.12)	<0.001	-0.20(-0.28, -0.11)	<0.001	-0.17(-0.26, 0.09)	<0.001

The crude model did not adjust for any covariates. Model Ⅰ adjusted for all covariates, including age, race, BMI, physical activity, family income, smoking status, drinking status, hypertension and diabetes mellitus. Model Ⅱ further adjusted for all other trace metals.

Abbreviations: Se, selenium; Mn, manganese; Co, cobalt; Ni, nickel; Mo, molybdenum; 95% CI: 95% confidence interval.

Table S7 The effects of iron status and inflammation biomarkers on the risk of anemia.

Iron status and inflammation biomarkers	Crude model		Model Ⅰ		Model Ⅱ	
	OR (95% CI)	*P* value	OR (95% CI)	*P* value	OR (95% CI)	*P value*
Ln-ferritin	0.93(0.91, 0.95)	<0.001	0.93(0.91, 0.94)	<0.001	0.94(0.92, 0.96)	<0.001
Albumin	0.97(0.97, 0.98)	<0.001	0.98(0.97, 0.98)	<0,001	0.98(0.98, 0.99)	<0.001
Ln-hsCRP	1.03(1.01, 1.04)	<0.001	1.03(1.01,1.04)	0.002	1.03(1.01, 1.04)	0.003

The crude model did not adjust for any covariates. Model Ⅰ adjusted for all covariates, including age, race, BMI, physical activity, family income, smoking status, drinking status, hypertension and diabetes mellitus. Model Ⅱ further adjusted for all trace metals.

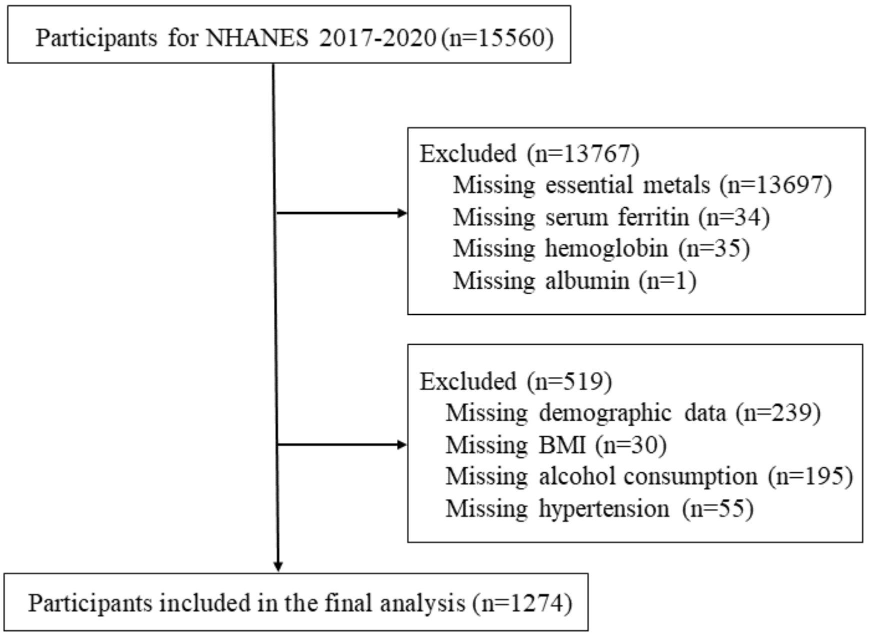

Figure S1 Flowchart of participants included in this study. NHANES, National Health and Nutrition Examination Survey; BMI, body mass index; demographic data included age, sex, race, family income levels.

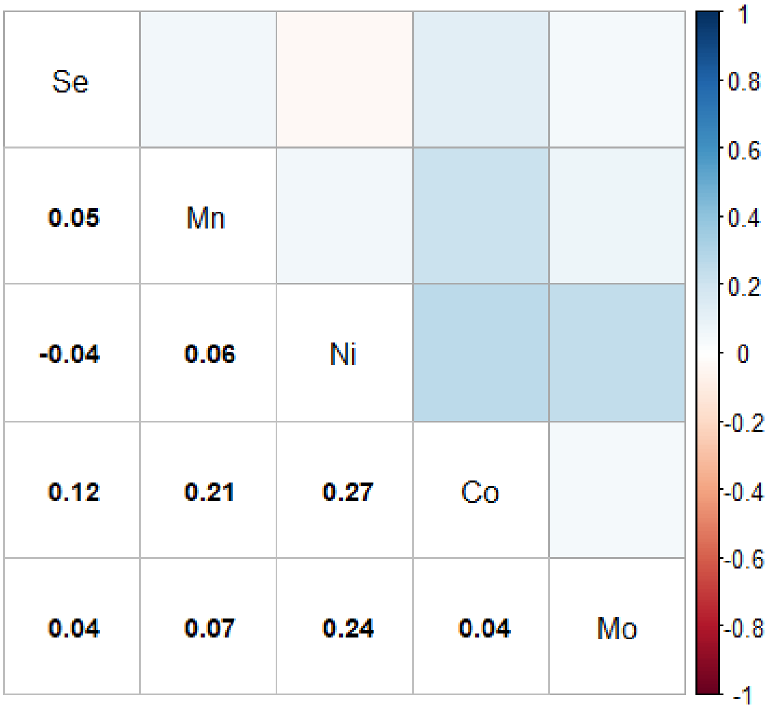

Figure S2 Spearman correlations among the five trace metals. Se, selenium; Mn, manganese; Co, cobalt; Ni, nickel; Mo, molybdenum.

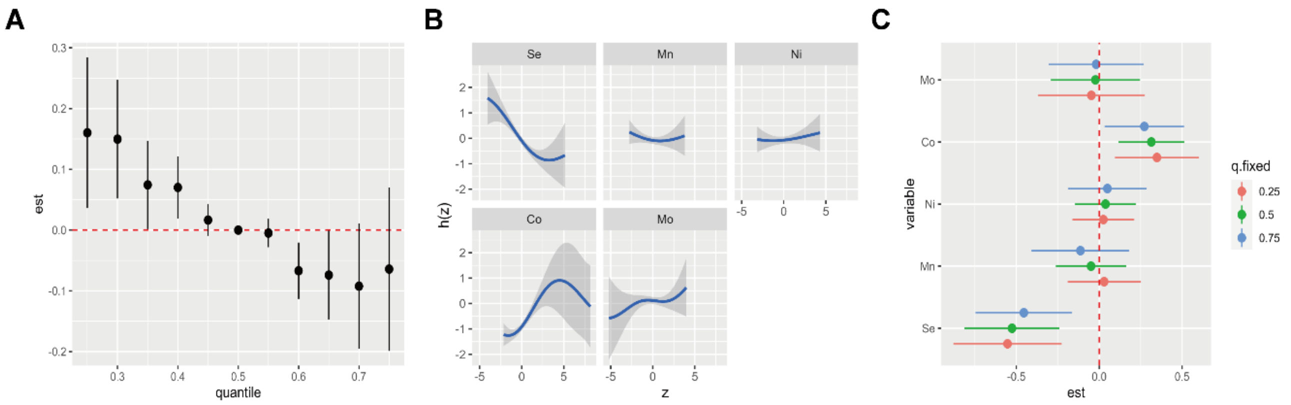

Figure S3 The associations between trace metals with anemia identified by the BKMR model in females. (A) Overall associations of the trace metal mixture with risk of anemia at increasing percentiles compared to medians. (B) Univariate exposure-response function between individual trace metal with the risk of anemia when fixed other metals at corresponding 50th percentiles. (C) Single-exposure effect of individual trace metal for an IQR increase on risk of anemia, when other metals were fixed at their 25th, 50th, or 75th percentiles. The model was adjusted for all covariates except for sex.

Abbreviations: Se, selenium; Mn, manganese; Co, cobalt; Ni, nickel; Mo, molybdenum.

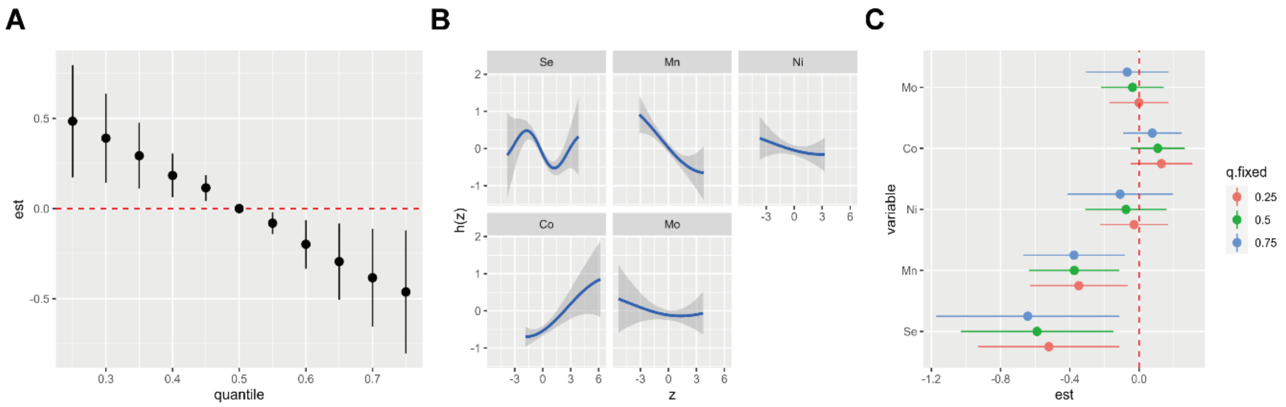

Figure S4 The associations between trace metals with anemia identified by the BKMR model in males. (A) Overall associations of the trace metal mixture with risk of anemia at increasing percentiles compared to medians. (B) Univariate exposure-response function between individual trace metal with the risk of anemia when fixed other metals at corresponding 50th percentiles. (C) Single-exposure effect of individual trace metal for an IQR increase on risk of anemia, when other metals were fixed at their 25th, 50th, or 75th percentiles. The model was adjusted for all covariates except for sex.

Abbreviations: Se, selenium; Mn, manganese; Co, cobalt; Ni, nickel; Mo, molybdenum.
